# A Review of the Literature on the Endocrine Disruptor Activity Testing of Bisphenols in *Caenorhabditis elegans*

**DOI:** 10.3390/jox16010007

**Published:** 2026-01-04

**Authors:** Patrícia Hockicková, Alžbeta Kaiglová, Marie Korabečná, Soňa Kucharíková

**Affiliations:** 1 Department of Laboratory Medicine, Faculty of Health Care and Social Work, Trnava University in Trnava, Univerzitné Námestie 1, 918 43 Trnava, Slovakia; patricia.hockickova@tvu.sk (P.H.); alzbeta.kaiglova@truni.sk (A.K.); marie.korabecna@lf1.cuni.cz (M.K.); 2 Institute of Biology and Medical Genetics, First Faculty of Medicine, Charles University and General University Hospital in Prague, Albertov 4, 128 00 Prague, Czech Republic

**Keywords:** endocrine disruptors, EDC, bisphenols, *Caenorhabditis elegans*, toxicity, toxicological endpoints, reproductive toxicity, neurotoxicity, oxidative stress, alternative toxicity testing, high-throughput screening

## Abstract

Endocrine disruptors, including bisphenol A, S, AF, and F, have been demonstrated to exhibit endocrine-disrupting activity. This phenomenon has been associated with a variety of health problems, including (but not limited to) neurological and reproductive disorders. Given the potential hazards, it is essential to have effective tools to assess their toxicity. The nematode *Caenorhabditis elegans* has become a widely used model organism for studying bisphenols because of its genetic simplicity and the conservation of its fundamental biological processes. This review article summarizes current knowledge of bisphenol toxicity and the use of the model organism *C. elegans* as a high-throughput system for investigating the toxicological profiles of BPA and its emerging alternatives. Furthermore, we highlight the specific methodologies for assessing the toxic effects of bisphenols in *C. elegans*. While highlighting its advantages, we critically discuss its limitations, including the absence of specific metabolic organs, which constrain direct extrapolation to mammalian systems. Based on available evidence, we conclude that *C. elegans* serves as an essential bridge between in vitro assays and mammalian models, offering a powerful platform for the early hazard identification and mechanistic screening of bisphenol analogues.

## 1. Endocrine-Disrupting Chemicals: A Significant Concern for Living Organisms

The World Health Organization (WHO) defines endocrine-disrupting chemicals (EDCs) as “exogenous substances or mixtures that alter the function of the endocrine system, resulting in adverse effects on the intact organism, its progeny, or populations” [1]. Currently, more than 1000 chemicals are recognized as EDCs, including industrial chemicals, pesticides, metals, and organometallics. Most EDCs are organic, lipophilic chemicals that accumulate in adipose tissue [2]. Human exposure occurs primarily through the ingestion of EDC-contaminated food and beverages, but may also occur via inhalation and dermal absorption. For many EDCs, dietary intake accounts for over 90% of the total exposure [2]. These chemicals exert a significant influence on human health by interacting with steroid hormone and neurotransmitter receptors, disrupting enzymatic pathways, or impairing hormone synthesis, transport, and distribution [3,4,5,6,7]. These disruptions are linked to reproductive disorders in women, including endometriosis, uterine fibroids, polycystic ovary syndrome (PCOS), ovarian failure, and infertility [8]. In men, EDCs have been associated with an increased risk of testicular cancer, undescended testicles, reduced serum testosterone levels, and poor sperm quality [9]. Prenatal exposure has been linked with cancer, autism, diabetes, infertility, attention-deficit/hyperactivity disorder (ADHD), cryptorchidism, hypospadias, and reduced anogenital distance in children [9,10,11]. Additionally, EDCs contribute to metabolic disorders such as obesity, type 2 diabetes mellitus, and cardiovascular complications [12,13].

Among these chemicals, bisphenols- such as bisphenol A (BPA), bisphenol S (BPS), bisphenol F (BPF), and bisphenol AF (BPAF)-are of particular concern [4,5,6,7]. Widely used in the manufacture of various plastic products, they are known for their toxicity and their ability to migrate from food-contact materials into food and the environment [6,14].

This review summarizes the most recent literature on the deleterious effects of bisphenols on human health. It provides a comprehensive overview of the current knowledge regarding the use of the model organism *Caenorhabditis elegans* in the toxicity testing of EDCs, with a particular focus on BPA and its alternatives, namely BPS, BPF, and BPAF.

### Bisphenols

BPA is an organic, synthetic compound used in the manufacturing of plastic packaging for food and beverages, dental sealants, and coatings for the lining of aluminum food and beverage cans [15]. Global production reached 7.72 million metric tons and is expected to increase [16]. Due to its mass production and wide range of uses, BPA is ubiquitous, entering the environment through production processes and through material degradation. The most common route by which BPA enters food and water is through the release from plastic packaging [17,18]. Despite its widespread use, this substance can accumulate in the human body and cause a range of adverse health effects. BPA is a well-characterized endocrine disruptor associated with numerous endocrine and metabolic disorders. Epidemiological evidence suggests a correlation between BPA exposure and reproductive disorders, including infertility [19], premature puberty, hormone-dependent tumors [20], PCOS [21], endometriosis [22], and impact on testicular cells (germ cells, Sertoli cells, and Leydig cells) [23,24]. Neurotoxic effects involve behavioral and cognitive impairments in young children [25,26,27] and impaired neurodevelopment [28]. Furthermore, data associate prenatal exposure with impaired fetal development [28,29], premature delivery, reduced placental weight, and preterm birth [30,31]. Metabolic effects include associations with obesity; higher BPA levels in urine or serum correlate with increased adiposity, and experimental models support BPA’s role as an obesogen [32,33]. Additionally, BPA exposure has been linked to immunotoxic [34] and genotoxic outcomes [35]. Recent integrative analyses have further emphasized that BPA and its analogues exert coordinated effects across the immuno-neuro-endocrine network, supporting a system-level model of bisphenol toxicity rather than isolated receptor-mediated mechanisms [36].

These are the key reasons why numerous countries, including the United States, Canada, China, and the European Union, have enacted legislation and regulations that limit or prohibit the use of BPA. As a consequence of these regulatory measures, BPA has been replaced with its chemical analogues, including BPS, BPF, BPAF, tetrabromobisphenol A (TBBPA), bisphenol AP (BPAP), bisphenol B (BPB), and bisphenol Z (BPZ) [37]. While these substitutes appear to offer considerable promise, their potential for adverse effects on human health is outlined below.

BPS represents one of the most widely used substitutes for BPA. Plastic products labeled “BPA-free” are frequently manufactured with the addition of BPS. The annual global production of BPS has reached approximately 44.6 thousand metric tons by 2020, and analysts have predicted that its production will increase by up to 11.52% by 2030 [38]. Despite its widespread use, evidence suggests that BPS can cross biological barriers, accumulate in tissues, and exert endocrine-disrupting effects similar to or stronger than those of BPA. Experimental studies and epidemiological data indicate that BPS exposure is associated with a range of biological effects across multiple systems. Its endocrine and reproductive effects include oxidative stress, impaired hormonal balance [39], delayed puberty, reduced ovarian weight, altered sex hormone levels in plasma [40], and altered morphology of the testicular epithelium, resulting in decreased testosterone levels in plasma [41]. Metabolic disruptions are evidenced by altered glucose metabolism and type 1 diabetes [42]. Developmental outcomes encompass reduced birth length, birth weight, and ponderal index [43,44], as well as spontaneous abortion [45]. Exposure of mammals to BPS adversely impacts the nervous system, leading to various neurobehavioral dysfunctions [27,46]. At a cellular level, BPS exposure has been linked to DNA damage [47] and immune system dysregulation [34].

Another frequently used analogue is BPF, which is employed in the production of a plethora of materials, including epoxy resins and coatings for a variety of purposes, such as the fabrication of water pipes, paints, food container linings, and dental sealants [48]. Similar to BPA, BPF enters the body primarily through the oral route, after which it is distributed throughout the body. Toxicokinetic studies confirm its ability to cross the placental membrane, evidenced by its detection in various biological matrices, including urine [49,50], breast milk [51], and blood [52]. Despite its broad usage, the toxicological profile of BPF remains incompletely characterized. The ongoing investigation into living organisms has identified a range of biological effects that warrant further investigation. Research suggests that exposure to BPF exerts reproductive toxicity, including hormonal imbalance, reduced sperm quality [53], impaired spermatogenesis, testicular morphology [54], altered ovarian function [55], and adverse pregnancy outcomes [56]. In addition, prenatal and postnatal exposure have been linked to fetal growth restriction, neurological [57,58,59], and metabolic disorders [56,58]. Cardiometabolic pathologies include obesity [60], altered weight [61], liver and kidney dysfunction, angina pectoris, stroke, and congestive heart failure [59,62,63,64,65,66], alongside skeletal effects [67].

A fluorinated derivative of BPA, utilized with lower frequency, is known as BPAF. It is employed in the production of plastic items, including polycarbonate copolymers, food-contact polymers, and electronic materials [68]. This substance has been classified as a reproductive toxicant due to its high estrogenic potency [69]. Compared to BPA, in vivo assays demonstrate that BPAF has a stronger affinity for estrogen receptors [70]. Experimental evidence indicates that BPAF induces reproductive toxicity [71,72,73,74,75]. Moreover, studies have revealed transgenerational effects [76], neurotoxic effects [77,78], and associations with obesity, reduced insulin sensitivity, and metabolic dysfunction [79,80,81]. Furthermore, experimental evidence suggests that these systemic effects are mediated at the cellular level by oxidative stress [82].

**Figure 1:**
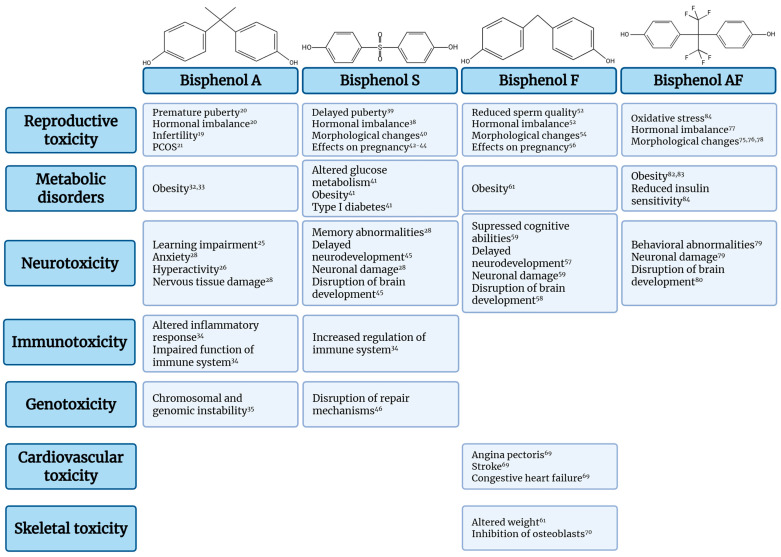
Overview of reported adverse health effects associated with Bisphenol A (BPA) and analogues (BPS, BPF, BPAF). The diagram summarizes toxicity effects. The effects listed are restricted to those showing statistical significance (*p* < 0.05) in epidemiological cohorts or experimental models [19,20,21,25,26,28,32,33,34,35,38,39,40,41,42,43,44,45,46,52,54,56,57,58,59,61,69,70,75,76,77,78,79,80,82,83,84].

The chemical structures, toxicity profiles, and negative effects of bisphenol A, S, F, and AF observed in humans and animal models are depicted in Figure 1. Importantly, many of these adverse outcomes, encompassing reproductive, neurodevelopmental, metabolic, immune, and multigenerational effects, reflect conserved biological processes that can be interrogated using defined mechanistic endpoints in *C. elegans*. These cross-species correspondences are summarized in Table 1 (see Section 2.1 for details).

## 2. The Nematode *Caenorhabditis elegans* as a Model Organism

Toxicity testing of chemicals is realized with the assumption that the information obtained in a specific model will be relevant for other biological systems. Mammalian models are still considered the gold standard for toxicity testing due to the high conservation of developmental pathways, physiological processes, and organ systems between mammals and humans. However, it is crucial to recognize that no experimental model is without constraints, and results obtained in any system may not fully predict complex population-level human responses [83]. The use of mammalian model organisms in scientific studies can be expensive and time-consuming [84]. In recent decades, alternative toxicological tools have been developed to complement traditional mammalian testing and to improve screening efficiency and mechanistic insight for environmental agents. These alternatives support the principles of reduction, refinement, and partial replacement (3Rs) rather than the complete replacement of mammalian models, and have promoted the increasing use of the invertebrate models, including *Caenorhabditis elegans*, as valuable screening and mechanistic tools [85,86].

*Caenorhabditis elegans* (*C. elegans*) is a free-living, non-pathogenic nematode that reaches a length of 1 mm at the adult stage. The nematode’s translucent body allows for the direct observation of cellular and biological processes, making it particularly useful for studies of EDCs, including bisphenols. The life cycle of the nematode takes approximately 3–4 days and consists of embryonic development, larval stages L1–L4, and the adult stage. The duration of embryonic development is approximately 16 h at laboratory temperature. Postembryonic development begins with hatching in the presence of food [87]. If conditions are unfavorable, the nematode may enter the dauer larval stage at the end of the L2 developmental stage. This condition, which is induced by environmental factors such as pheromones (indicators of population density), the absence of food, or high temperature, results in arrested senescence, reduced motility, and cessation of food intake. If circumstances change, the nematode will complete this stage in an hour and initiate the consumption of food again. The nematode then enters the L3 larval stage, which persists for 12 h. It is during this stage that the most significant alterations occur in the reproductive system [88,89]. In the L4 larval stage, the nematodes begin to produce offspring for 2–3 days. Hermaphrodites are capable of producing around 300 offspring, whereas when mated with a male, they can produce 1200–1400 offspring. Following the fertile period, the adult nematode lives for approximately 10–15 days [90]. Together, these features enable rapid assessment of neurobehavioral, reproductive, and developmental endpoints across multiple generations, which is especially valuable for bisphenol toxicity testing.

The most commonly used strain in laboratory conditions is *C. elegans* N2. Nematodes are cultured on Nematode Growth Medium (NGM) plates, with *Escherichia coli* OP50 (a uracil auxotroph) used as the food source. Growth of this bacterium is limited on the plates, which allows for observing the nematodes in an easier and better way [91]. In laboratory conditions, it is possible to maintain large populations of nematodes in 96-well microtiter plates, allowing simultaneous testing of multiple compounds or mixtures at different concentrations and enabling high-throughput approaches suitable for bisphenol screening [83].

This nematode possesses several characteristics that make it a powerful model for biological research. It can be easily and relatively inexpensively maintained, has a short life cycle, and has a large number of progeny. Due to its small size and transparent body, we can directly visualize cellular processes and whole-organism responses simultaneously. In 1998, the entire genome of the nematode was successfully sequenced, being the first sequenced genome of a multicellular organism [92]. These findings have enabled the development of techniques to manipulate and study *C. elegans* at the molecular level [93], including the creation of transgenic reporter strains for in vivo monitoring of oxidative stress, DNA damage, and gene expression changes. The nematode is also an excellent experimental organism for studying molecular and cellular aspects of human diseases in vivo. It is believed that approximately 42% of human genes have an ortholog in the *C. elegans* genome. The use of this nematode in genetic analyses provides the advantage of genetic screens that allow relatively rapid identification of proteins and molecular pathways that are involved in specific cellular processes [94].

*C. elegans* enables assays to be performed ranging from the whole-organism to the single-cell level. A common procedure in chemical testing consists of incubating nematodes in a medium containing a toxic substance at several concentrations. Following this, selected endpoints are monitored, which include biological parameters such as lethality, growth, movement, or reproduction. To identify oxidative stress, DNA damage, or changes in gene expression, molecular markers can also be employed [95]. The use of transgenic reporter strains allows mechanistic insights into stress-response pathways, facilitating the identification of molecular targets of bisphenols.

Because nematodes are highly sensitive to environmental exposure, they are a valuable tool in toxicological research for investigating the impacts of compounds, extracts, and nanomaterials [96,97]. Although *C. elegans* lacks specialized endocrine glands, hormone-like molecules are produced by various cells and tissues with other primary roles [98]. Nonetheless, conserved neuroendocrine pathways regulate dauer diapause, reproduction, and aging [99,100,101]. It is thought that vertebrate hormones such as steroids may also have endocrine functions in nematodes [102]. These pathways permit functional assessment of endocrine disruptors, with endpoints ranging from molecular responses to whole-organism effects. *C. elegans* is a valuable model organism utilized to investigate the impact of endocrine-active environmental contaminants [103]. Toxic effects applicable to humans have been identified through assays using nematode embryos. Unlike assays based on cultured cells, these assays offer the advantage of accounting for the complexity of the entire organism, revealing systemic effects of bisphenols while maintaining the ability to dissect molecular mechanisms. As a result, these tests can provide supplementary information to in vitro studies. However, no test using nematode embryos has been officially approved for use in assessing human risk [104].

**Figure 2:**
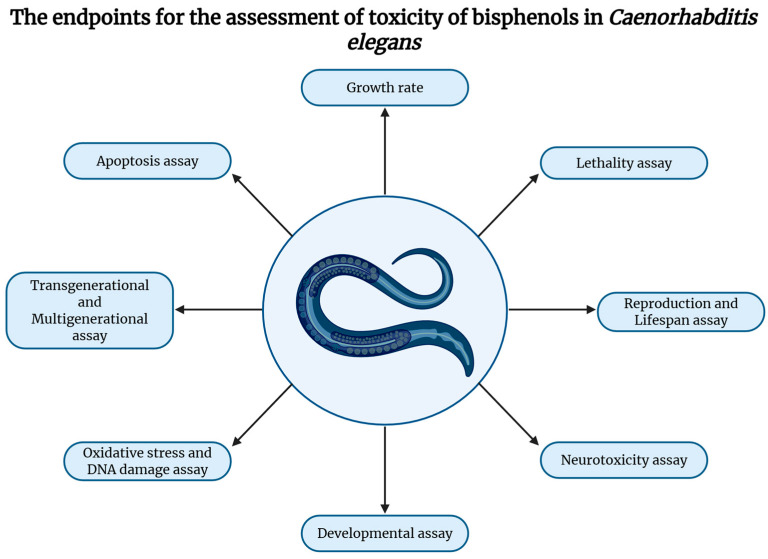
The evaluation of environmental pollutants’ toxicity using *Caenorhabditis elegans* as a model organism.

Overall, the toxicological assessment of environmental pollutants using *C. elegans* includes endpoints (described in more detail below) such as development, behavior, reproduction, reactive oxygen species (ROS) production, apoptosis, and stress response [93,103,105] (Figure 2). The combination of rapid generation time, genetic tractability, transparency, high fecundity, and compatibility with high-throughput formats makes *C. elegans* an efficient and mechanistically informative model for bisphenol and EDCs screening.

### 2.1. The Endpoints Employed for the Assessment of Toxicity of Bisphenols in C. elegans

#### 2.1.1. Lethality Assay

Lethality tests assess the mortality rates of nematodes, usually using concentration-response curves derived from short-term (several hours) exposure to chemicals at 20 °C, without food. Mortality is determined by stereomicroscopic inspection in order to enumerate the number of live and dead individuals following exposure. The characterization of nematode mortality can be achieved through either a 30-s observation period or by noting the absence of a response to a gentle stimulus provided by a small metal wire [106,107,108,109]. However, lethality outcomes are strongly influenced by experimental design factors, including nematode strain, exposure duration, solvent composition, and the method of mortality assessment (manual or automated), thereby constraining direct comparability across studies. Existing studies have shown that environmental contaminants, including bisphenols, significantly reduce nematode survival in a dose- and time-dependent manner. It has been demonstrated that BPA induces concentration-dependent lethality in *C. elegans* [110,111], with synergistic mortality observed in combined exposure to BPA and BPS [112,113]. Nevertheless, despite the frequent observation of clear dose–response patterns, many studies utilize exposure concentrations that exceed environmentally relevant levels, potentially limiting the extrapolation of findings to human health contexts. Bisphenol-induced lethality is closely associated with systemic oxidative stress, mitochondrial dysfunction, and cumulative cellular damage. Together, these processes compromise detoxification and repair mechanisms and culminate in organismal death.

#### 2.1.2. Growth Rate and Developmental Assay

The utilization of *C. elegans* as a model organism facilitates the examination of the impacts of environmental pollutants on growth and development due to its simple life cycle, transparency, and ease of culture. Growth assays typically involve exposing nematodes to test compounds at various larval stages. Following exposure, nematodes are cultured under standard conditions to facilitate growth and development. Thermal shocks are frequently used to straighten the worm body, facilitating exact measurements by allowing length and width to be determined precisely by image analysis software [110,114,115,116]. Multiple studies have demonstrated the effectiveness of this method in evaluating the effects of environmental pollutants on nematode growth. Exposure to BPA and BPS reduces the body length of the nematode [112,117,118,119,120], with similar effects reported for other bisphenols such as TBBPA [121]. Recognizing the inconsistencies in current research is essential, as several studies indicate an increase in body length following BPA exposure [110]. These divergent findings may be explained by differences in experimental conditions, including BPA concentration, exposure duration, developmental stage at exposure, temperature, nematode strain, and culture methods. The observed disparities underscore the complexity of the nematode’s response to chemical stresses and the necessity for additional research. Beyond growth, developmental toxicity can be assessed by periodically counting the number of individuals in each developmental stage or monitoring dauer larval formation as indicators of developmental stress [122]. Although dose-dependent effects are frequently observed, most studies employ supraphysiological bisphenol concentrations, underscoring the need to interpret these findings in the context of environmentally and human-relevant exposure levels. These impairments in growth and developmental progression appear to result from bisphenol-induced oxidative damage, metabolic dysregulation, and disruption of endocrine-regulated growth signaling—processes that collectively interfere with cellular proliferation, energy homeostasis, and somatic development.

#### 2.1.3. Reproduction and Lifespan Assay

The ability to reproduce is a key test for evaluating the potential of endocrine disruptors. In this regard, *C. elegans* is a highly suitable model organism for studying reproductive toxicity due to its highly differentiated, yet simple, reproductive system. It is well known that nematodes are mostly hermaphrodites, where both sperm and oocytes develop gradually from a common gonad [123]. To investigate the effects of environmental pollutants on *C. elegans*’ reproduction, researchers use a variety of methodologies. A common strategy is to compare the size and number of progeny produced by exposed worms with a control group [104,124]. This objective may be accomplished by transferring exposed adult worms to a Petri dish and enumerating all hatched progeny [125]. Alternatively, the fertility rate may be estimated using the total number of larvae at the end of the experiment. Another indicator of reproductive health is egg-laying behavior. This may be determined by placing adult worms in a new Petri dish and counting the number of eggs laid over a specific time period [87]. Exposure to numerous endocrine disruptors, such as BPA, BPS, and tetrachlorobisphenol A (TCBPA), has been shown to reduce offspring production, development, and brood size and increase embryonic and larval lethality [111,118,126,127,128]. In addition, different types of BPA analogs, such as bisphenol Y (BPY), BPZ, BPF, tetramethyl bisphenol F (TMBPF), and bisphenol TMC (BPTMC), exert comparable detrimental effects on the reproductive system of *C. elegans* [118,129,130]. These reproductive deficits appear to be mechanistically driven by oxidative stress-induced DNA damage, disruption of meiotic chromosome segregation, and activation of germline apoptosis, processes that collectively compromise embryonic viability. It should be noted that many studies employ high bisphenol concentrations, exceeding environmental exposure, which may amplify observed effects.

The evaluation of lifespan is another effective approach to examine the impacts of endocrine disruptors in *C. elegans*. The duration from the L4 larval stage to death is the traditional definition of nematode lifespan. To demonstrate this phenomenon, researchers subjected L4 larval stage worms to a test chemical and observed their survival over time. Nematodes are routinely transferred to fresh plates with sustenance and 5-fluorodeoxyuridine to inhibit reproduction. The survival rate is calculated by dividing the number of living nematodes by the total number of nematodes [115,131,132,133]. Recent studies have indicated that exposure of *C. elegans* to some endocrine disruptors, including BPA, BPF, BPS, and TMBPF, significantly diminished the nematode’s lifespan [117,118]. Decline in lifespan appears to be associated with accelerated accumulation of molecular damage, persistent redox imbalance, and impaired proteostasis, suggesting that bisphenol exposure contributes to premature functional decline and aging-related phenotypes.

#### 2.1.4. Transgenerational and Multigenerational Assay

The term “transgenerational and multigenerational toxicity” refers to the harmful effects of environmental pollutants on both the exposed generation and their progeny [134,135]. *C. elegans*, with its short life cycle and high fecundity, provides an effective model organism for such studies [135]. Existing data indicate that exposure to BPA may affect nematode reproduction across generations, potentially mediated by deregulation of repressive histone modifications [136]. In a similar vein, BPS exposure induces transgenerational behavioral and developmental alterations, including reduced head thrashes [117], decreased body length of offspring, and reproductive output over multiple generations [117,137]. Similarly, TBBPA elicits transgenerational toxicity, manifested as diminished locomotion activity [138], reduced lifespan, and survival rates across three generations [139]. These multigenerational effects are likely mediated by cumulative oxidative stress, DNA damage, and epigenetic modifications, which impair neuronal function, reproduction, and development in subsequent generations. Methodological differences, including the number of generations studied, timing of exposure, strain selection, and culture conditions, can lead to variable outcomes across laboratories.

#### 2.1.5. Neurotoxicity Assay

*C. elegans* is a widely used model for assessing the neurotoxic effects of environmental pollutants, with locomotion serving as a sensitive indicator of neuronal function. Given that neuronal function is highly dependent on energy balance and redox homeostasis, neurotoxicity often represents one of the earliest and most sensitive functional manifestations of systemic, bisphenol-induced cellular stress.

Behavioral assays typically measure head thrashes, rapid bending movements of the body, or sinusoidal body bends [140,141,142,143]. In these assays, synchronized young adult worms (day-1 adults) are commonly used [142,143]. Worms are transferred to a liquid medium (e.g., K-medium, M9 buffer, or S-medium) for thrashing/swimming assays [144] or may be assayed on agar plates for body bending assays [145]. After transfer, worms are allowed a brief acclimation period (approximately 30 s) before scoring begins. Head thrashes are quantified by counting the number of occurrences within a defined scoring window, often 30 s to 1 min, while body bends are similarly recorded over a set interval. Typically, 10–20 worms per replicate are measured, with multiple biological replicates (e.g., three independent experiments) to ensure statistical reliability [146]. An alternative test involves counting immobile worms, which are recorded when there is no movement following gentle stimulation with a platinum wire [147]. Locomotion assays can be scored manually by the observer or automatically using tracking software, depending on the experimental setup [144,148]. It should be noted that experimental conditions, including exposure duration, observation time, scoring methods (manual vs. automated), and stimulus intensity, are not fully standardized and may differ among studies, potentially contributing to variability in reported outcomes. Exposure to environmental pollutants, such as BPA, BPS, TBBPA, and TCBPA, significantly reduces locomotor activity, reflecting potential neurotoxicity [112,117,121,143,149,150,151]. These neurobehavioral deficits are linked to oxidative stress, DNA damage, and apoptotic processes in neuronal cells. Moreover, high bisphenol concentrations often used in these studies may exaggerate effects compared to environmental exposure levels.

Chemotaxis is a critical behavior for *C. elegans*, essential for foraging, mate location, egg laying, dauer larva formation, and evasion of pathogens and toxins. Chemotaxis tests reliably assess behavioral responses to the chemical stimuli. In a conventional experimental design, the worms are evenly distributed between a test substance and a control substance that is put on opposite sides of a Petri dish. Worms tend to move toward areas containing the test substance when they are attracted to it. In contrast, if they are repulsed, the worms will move away, resulting in a higher concentration of worms on the control side compared to the test side. With this approach, researchers can study and measure the chemotactic responses of *C. elegans* to various environmental stimuli. In addition to analyzing attraction or repulsion, researchers may also determine preference behavior between distinct compounds. This is accomplished by simultaneously introducing two unique attractants on opposing sides of the Petri dish, rather than one attractant and a control. The worm distribution is used to construct the Chemotaxis Index (CI), a calculated parameter that facilitates a comparative evaluation of the chemotaxis response to each option. This approach has been demonstrated to yield significant insights into the preferences of worms [103,114,152,153,154,155].

The pharynx of *C. elegans* demonstrates a pumping action that is similar to that of the mammalian heart and serves as a key indicator of feeding behavior. Pharyngeal pumping assays quantify feeding behavior by counting the number of pharyngeal contractions in a defined time period, often using age-synchronized nematodes to ensure uniformity with regard to size and developmental stage [156,157]. Alternative methods measure food consumption via changes in the bacterial food density in liquid cultures [158,159] or the ingestion of fluorescent microspheres [158]. Exposure to bisphenols, such as BPA and TBBPA, considerably reduces pharyngeal pumping rates, leading to decreased food intake and consequently causing adverse effects on general health [121,149,160]. Reduced pharyngeal pumping is likely a downstream effect of neuronal impairment caused by oxidative stress and DNA damage, demonstrating how neurotoxicity and general health are interconnected.

#### 2.1.6. Oxidative Stress and DNA Damage Assay

Oxidative stress, resulting from an imbalance between ROS production and the antioxidant defense of the body, is a common mechanism of environmental toxicant-induced toxicity. Elevated ROS levels can cause serious damage to proteins, lipids, and, most importantly, DNA, leading to observable phenotypes in *C. elegans*, such as reduced motility, decreased body length, and impaired development of offspring. Consequently, oxidative stress appears to constitute a central upstream pathway through which bisphenols mediate toxicity across downstream endpoints, including developmental delays, neurobehavioral dysfunction, reproductive impairments, apoptosis, and reduced longevity. As demonstrated in the existing literature, there are several different methods to measure oxidative stress in nematodes; for example, lipofuscin measurements or utilization of fluorescent dyes like 2′,7′-dichlorofluorescin diacetate. Moreover, qPCR of GFP reporters induced by oxidative stress-responsive genes offers further insights into the cellular response [112,142,149,161,162]. It has been displayed that exposure to environmental contaminants results in significant DNA damage. To investigate these effects in *C. elegans*, qPCR, comet assay, and transgenic strains containing DNA damage and repair reporters can be employed [143,161,163]. It has been previously documented that BPA can cause oxidative stress in *C. elegans*, resulting in increased ROS levels. This elevation inhibits the repair of DNA double-strand breaks during meiosis, thus negatively affecting reproduction, the nervous system [126,143,164], and accelerating the aging process [165]. BPA can also cause chromosomal abnormalities and double-strand breaks in meiotic DNA in *C. elegans* [166]. Similar effects have been observed with BPS [117] and TBBPA [167].

#### 2.1.7. Apoptosis Assay

Apoptosis in *C. elegans* is commonly assessed by evaluating cellular morphology and DNA fragmentation, using fluorescent dyes from transgenic strains. A common technique employed in this field involves the use of the essential dye acridine orange. This dye exhibits a vivid green, fluorescent color and has the ability to penetrate the nucleus [168]. During the experimental procedure, the nematodes are submerged in acridine orange for two hours following exposure to a toxicant, which facilitates effective dye absorption. Apoptotic cells may be identified using an inverted fluorescence microscope with specific excitation and emission wavelengths following their recovery in a Petri dish. Apoptotic cells have enhanced DNA fragmentation, accompanied by a characteristic yellow or yellow-orange fluorescence. On the other hand, healthy cells maintain their green fluorescence [112]. An alternative technique involves the utilization of SYTO12, a fluorescent stain, which is applied to *C. elegans* for 1.5 h at room temperature. After staining, the nematodes are transferred to seeded NGM plates with *E. coli* OP50 for 30 min to remove any residual staining. Apoptotic cells can later be identified using a fluorescence microscope with a red filter [169]. Transgenic strains that express ced-1, which encodes a transmembrane protein required for ingesting germ cell corpses, have been employed in research to target germ cell death. When compared to acridine orange staining, this approach offers better results [170]. Exposure to bisphenols, including BPA, BPF, BPS, BPY, BPZ, and TBBPA, significantly enhances DNA fragmentation and apoptosis [109,112,129,139], with synergistic effects observed during combined exposures [113,171]. Apoptosis serves as a mechanistic mediator connecting oxidative stress and DNA damage to functional outcomes such as reproductive toxicity, neurobehavioral deficits, and reduced lifespan. Differences in reporter strains, staining protocols, and exposure duration contribute to variability in results. As with other endpoints, high bisphenol concentrations commonly used in experiments may exaggerate apoptotic responses compared to environmentally relevant levels.

To synthesize these findings and evaluate the translational value of the nematode model, Table 1 provides a comparative overview of the conserved mechanistic pathways identified in *C. elegans* against known mammalian outcomes. Collectively, these endpoints provide a comprehensive, multi-level assessment of bisphenol toxicity in *C. elegans*, encompassing developmental, reproductive, neurobehavioral, and molecular effects. However, observed outcomes can vary substantially depending on experimental conditions, including nematode strain, exposure concentration and duration, developmental stage at exposure, and assay methodology, highlighting the need for careful standardization and cautious extrapolation when interpreting results across studies and to higher organisms.

**Table 1:** Cross-species comparison of key bisphenol-induced mechanistic pathways and their functional readouts in *C. elegans.* This synthesis highlights the concordance between mammalian outcomes (human epidemiological data and rodent models) and specific *C. elegans* toxicity endpoints described in this review.

Bisphenol-Relevant Mechanistic Pathway	Representative Mammalian/Human Outcomes Reported for BPA and Analogues	Corresponding *C. elegans* Endpoints Used for Bisphenol Toxicity	Translational Relevance and Limitations
Oxidative stress and mitochondrial dysfunction	BPA, BPS, BPF, and BPAF increase ROS production and antioxidant defenses [38,44,48,56]	ROS-sensitive dyes, reporter strains, oxidative-stress responsive GFP strains, survival [115,130,146,147,153,165,166,168]	Central, conserved mechanism for bisphenol toxicity, high sensitivity in nematodes. Dose–response extrapolation requires caution due to metabolic differences
Reproductive and endocrine disruption	BPA, BPS, BPF, and BPAF are associated with infertility [19], PCOS [21], reduced sperm quality [40,52,53,54], altered ovarian function [39,55], and hormone homeostasis [52,72,73]	Brood size, egg-laying rate, germline apoptosis, developmental progression [114,121,130,131,132,133,134]	Conserved germline biology supports translational relevance; however, nematodes lack vertebrate gonadal steroid hormones
Neurobehavioral toxicity	Neurodevelopmental impairment [28,45,57], cognitive and behavioral deficits [25,26,27] after bisphenol exposure	Thrashing and body-bend frequency, chemotaxis behavior, pharyngeal pumping [115,120,124,147,153,154,155,164]	Functional neurotoxicity is robustly captured; neurotransmitter-specific effects may need complementary molecular analysis
Metabolic dysregulation and obesogenic effects	Bisphenol exposure is associated with increased adiposity [32,33,61,82], insulin resistance [41], and metabolic syndromes [63,64,83]	Growth rate, body bend, lifespan, developmental timing [115,120,121,122,123]	Insulin/IGF-1 signaling is conserved; nematode lipid storage reflects energy homeostasis rather than true adiposity
DNA damage	DNA strand breaks, chromosomal instability, and impaired DNA repair [35,38,46]	Comet assay, DNA damage reporter strains, qPCR, meiotic chromosome integrity [130,147,165,167,168]	High mechanistic concordance for DNA damage pathways, cancer risk cannot be directly modeled
Transgenerational and epigenetic effects *	BPAF is associated with inherited reproductive, developmental, and behavioral alterations [30,31,42,43,79]	Multigenerational brood size, lifespan, and behavioral assays, reporter strains [120,140,141,142,143]	*C. elegans* is particularly powerful for tracking inheritance across generations, though epigenetic complexity is reduced compared to mammals

* Transgenerational and epigenetic effects: *C. elegans* endpoints demonstrate true multigenerational inheritance; mammalian citations primarily refer to prenatal/developmental exposure outcomes due to the long generation time of vertebrates.

## 3. Limitations of Using *C. elegans*

**Figure 3:**
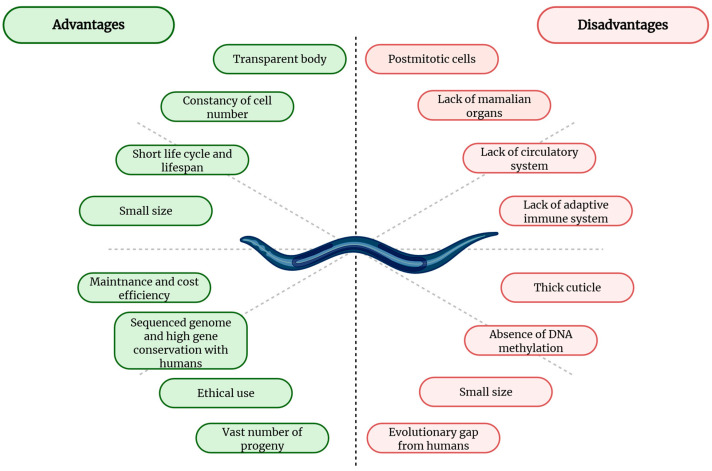
The pros and cons of using the *Caenorhabditis elegans* model for bisphenol toxicity testing.

*C. elegans* serves as an important model organism for investigating environmental and genetic toxicity, owing to its well-defined genetics, short lifespan, and ease of culture. In order to ensure accurate interpretation and application of results concerning EDCs and bisphenol-associated risks to human health, it is essential to acknowledge not only the advantages but also the limitations of the model (Figure 3).

While *C. elegans* offers a valuable high-throughput platform for identifying potential EDCs, it is important to acknowledge the limitations of this model regarding toxicokinetic relevance to humans. The primary limitation of nematodes is their simpler biological organization; specifically, *C. elegans* lacks organs analogous to the human liver and kidneys, which are primary sites for xenobiotic metabolism and excretion [83,172]. Consequently, the bioaccumulation and metabolic activation or detoxification of bisphenols in nematodes may differ from mammalian pathways [173]. Therefore, findings from *C. elegans* should be interpreted as potential indicators of toxicity that warrant further verification in mammalian systems or epidemiological studies. Adult *C. elegans* somatic cells lack the ability to replicate (postmitotic), making them unsuitable for stem cell and tumor research [174], which limits their applicability for evaluating hormone-dependent tumorigenesis, a key concern associated with BPA exposure. In addition, the absence of complex organ systems such as vertebrate-like brain, circulatory system, and adipose tissue makes them less useful for studying systemic endocrine, neuroendocrine, and metabolic effects of bisphenols [96,175,176]. This limitation is particularly relevant given evidence that bisphenols act through integrated immune-neuroendocrine interactions rather than isolated pathways, underscoring the need for cautious extrapolation [36].

Additionally, *C. elegans* lacks specialized endocrine glands and key vertebrate hormone receptors, including the estrogen receptor. While nematodes produce hormone-like molecules that regulate reproduction, development, and stress responses through conserved signaling pathways, the absence of these receptors limits the organism’s ability to fully model the estrogenic effects of bisphenols [177]. Although *C. elegans* possesses an innate immune system, the lack of an adaptive immune and myelination system restricts its application in immunotoxicity studies [178]. This is a critical limitation, given that immune dysregulation is a primary target of bisphenol toxicity [179,180]. Regulatory assessments, including those by EFSA, emphasize immune endpoints as particularly sensitive for evaluating safe bisphenol exposure [181]. Therefore, immunotoxicity findings in nematodes should be interpreted cautiously and confirmed in mammalian models. Researchers occasionally utilize phenologs, organ-like functional analogues in simpler organisms that mimic the physiological or cellular role of mammalian organs, such as the pharynx serving as a cardiac model [182,183], to partially bridge the gap in modeling bisphenol-induced specific effects.

Nonetheless, the considerable evolutionary disparity between nematodes and humans may result in variable responses from the model. The small size of nematodes can pose considerable challenges in the execution of specific toxicity assessments. Moreover, the fact that bacterial culture serves as a food source for nematodes raises concerns regarding potential metabolic changes that could affect test results [178], as bacterial biotransformation may alter the bioavailability and internal dose of bisphenols, influencing observed toxicological endpoints. The thick cuticle has been demonstrated to reduce the amount of test substances inside the nematode in comparison to the amount applied externally. This is particularly important for bisphenols such as BPA, BPF, and BPAF, as their lipophilicity may limit uptake in Wild-Type nematodes. Consequently, mutant strains with altered cuticle are often used to ensure accurate internal exposure and assessment of bisphenol toxicity [178,184].

DNA methylation, a regulatory mechanism that affects gene expression in mammals, is absent in *C. elegans*. Variations in this aspect may result in differences in organismal responses to certain toxins [141]. This represents an important limitation for bisphenol research, as BPA and its analogues are known to induce epigenetic modifications and transgenerational effects in mammalian systems. Furthermore, the human or mammalian genome is significantly more complex than the nematode’s genome. This genomic disparity may affect the capacity of *C. elegans* to identify or respond to particular toxicants, including bisphenols [96].

It is noteworthy that certain molecular pathways present in humans may not exist in *C. elegans*. This may complicate the identification of toxic effects observed in in vivo assays [83,96]. It is possible for even small changes in the environment or the mishandling of cultures to elicit adaptive responses in *C. elegans*, which may consequently lead to different test outcomes across generations. This restriction may be diminished through the use of appropriate nematode handling techniques and the option of using different phenotypes [83].

Nevertheless, *C. elegans* remains a highly valuable complementary model organism in the fields of environmental and genetic toxicity research, including bisphenol testing. To guarantee accurate data interpretation and draw significant conclusions about potential hazards to human health, it is essential to understand its limitations. The identification of these constraints and the use of appropriate strategies allows researchers to leverage the benefits of *C. elegans* while minimizing the risk of misinterpretations. When integrated with mammalian data, this model provides critical mechanistic insights and early hazard identification, thereby supporting human health risk assessment and evidence-based regulatory decision-making for emerging bisphenol substitutes.

## 4. Conclusions

The critical evaluation of the literature confirms that the transition from BPA to structural analogues, such as BPS, BPF, and BPAF, does not necessarily mitigate health risks, as these substitutes exhibit comparable, and in some cases more potent, toxicity profiles. The available evidence synthesized in this review underscores that *C. elegans* is not merely a screening tool, but a sophisticated model capable of elucidating the conserved molecular mechanisms underlying bisphenol toxicity, particularly oxidative stress, DNA damage, and apoptosis. These cellular disturbances consistently manifest as downstream adverse effects across the bisphenol class, driving reproductive failure, neurodevelopmental delays, and multigenerational impairments.

While the utility of *C. elegans* in high-throughput toxicology is robust, a balanced interpretation of these findings requires an acknowledgment of the model’s intrinsic limitations. Specifically, the absence of mammalian-like metabolic organs, such as the liver and kidneys, implies that the bioaccumulation and biotransformation pathways in the nematode may not fully reflect human toxicokinetics. Furthermore, the lack of an adaptive immune system and specific steroid receptors necessitates caution when extrapolating immunotoxic and estrogen-receptor-mediated outcomes to vertebrates. Therefore, data derived from this model should be viewed as a crucial prioritization signal within a broader tiered testing strategy, rather than a direct replacement for mammalian safety assessments.

Looking forward, the field must move beyond simple descriptive toxicity studies. Future research should leverage the genetic tractability of *C. elegans* to map specific receptor-mediated pathways for emerging bisphenols, filling the knowledge gap. From a regulatory perspective, we advocate for the integration of *C. elegans* assays into pre-market screening protocols for new chemical entities. Implementing comparative toxicity ranking using multi-endpoint assays, encompassing lethality, reproduction, and neurobehavior, would provide a rapid, cost-effective method to identify “regrettable substitutions” before widespread industrial application. By combining high-throughput in vivo screening in nematodes with targeted mammalian validation, researchers can accelerate the identification of safer alternatives, ultimately supporting more evidence-based environmental health policies.

## Data Availability

No new data were created or analyzed in this study. Data sharing is not applicable to this article.

## Abbreviations

The following abbreviations are used in this manuscript:

WHO	World Health Organization
EDC	Endocrine-Disrupting Chemicals
ADHD	Attention-Deficit/Hyperactivity Disorder
BPA	Bisphenol A
BPS	Bisphenol S
BPF	Bisphenol F
BPAF	Bisphenol AF
PCOS	Polycystic Ovarian Syndrome
TBBPA	Tetrabromobisphenol A
BPAP	Bisphenol AP
BPB	Bisphenol B
BPZ	Bisphenol Z
NGM	Nematode Growth Medium
ROS	Reactive Oxygen Species
TCBPA	Tetrachlorobisphenol A
TMBPF	Tetramethylbisphenol F
BPTMC	Bisphenol TMC
CI	Chemotaxis Index
qPCR	Quantitative Polymerase Chain Reaction
GFP	Green Fluorescent Protein
BPY	Bisphenol Y

## References

[1] World Health Organization Global Assessment on the State of the Science of Endocrine Disruptors. https://iris.who.int/handle/10665/67357.

[2] Yilmaz B., Terekeci H., Sandal S., Kelestimur F. (2019). Endocrine Disrupting Chemicals: Exposure, Effects on Human Health, Mechanism of Action, Models for Testing and Strategies for Prevention. Rev. Endocr. Metab. Disord..

[3] Ho V., Pelland-St-Pierre L., Gravel S., Bouchard M.F., Verner M.A., Labrèche F. (2022). Endocrine Disruptors: Challenges and Future Directions in Epidemiologic Research. Environ. Res..

[4] Diamanti-Kandarakis E., Bourguignon J.P., Giudice L.C., Hauser R., Prins G.S., Soto A.M., Zoeller R.T., Gore A.C. (2009). Endocrine-Disrupting Chemicals: An Endocrine Society Scientific Statement. Endocr. Rev..

[5] Thomas Zoeller R., Brown T.R., Doan L.L., Gore A.C., Skakkebaek N.E., Soto A.M., Woodruff T.J., Vom Saal F.S. (2012). Endocrine-Disrupting Chemicals and Public Health Protection: A Statement of Principles from The Endocrine Society. Endocrinology.

[6] La Merrill M.A., Vandenberg L.N., Smith M.T., Goodson W., Browne P., Patisaul H.B., Guyton K.Z., Kortenkamp A., Cogliano V.J., Woodruff T.J. (2019). Consensus on the Key Characteristics of Endocrine-Disrupting Chemicals as a Basis for Hazard Identification. Nat. Rev. Endocrinol..

[7] Monisha R.S., Mani R.L., Sivaprakash B., Rajamohan N., Vo D.V.N. (2022). Remediation and Toxicity of Endocrine Disruptors: A Review. Environ. Chem. Lett..

[8] Hassan S., Thacharodi A., Priya A., Meenatchi R., Hegde T.A., R T., Nguyen H.T., Pugazhendhi A. (2024). Endocrine Disruptors: Unravelling the Link between Chemical Exposure and Women’s Reproductive Health. Environ. Res..

[9] Thacharodi A., Hassan S., Acharya G., Vithlani A., Hoang Le Q., Pugazhendhi A. (2023). Endocrine Disrupting Chemicals and Their Effects on the Reproductive Health in Men. Environ. Res..

[10] De Oliveira I.V.M., De Albuquerque F.M., De Jesus Fernandes A., Berti Zanella P., Alves Silva M. (2025). Prenatal Exposure to Bisphenol-A and Neurocognitive Changes in Children Aged 2 to 5 Years: A Systematic Review. Rev. Environ. Health.

[11] Zhang J., Yuan M., Liu Y., Zhong X., Wu J., Chen W. (2025). Bisphenol A Exposure and Neurodevelopmental Disorders and Problems in Children under 12 Years of Age: A Systematic Review and Meta-Analysis. J. Hazard. Mater..

[12] McAllister E.J., Dhurandhar N.V., Keith S.W., Aronne L.J., Barger J., Baskin M., Benca R.M., Biggio J., Boggiano M.M., Eisenmann J.C. (2009). Ten Putative Contributors to the Obesity Epidemic. Crit. Rev. Food Sci. Nutr..

[13] Ahn C., Jeung E.B. (2023). Endocrine-Disrupting Chemicals and Disease Endpoints. Int. J. Mol. Sci..

[14] Tumu K., Vorst K., Curtzwiler G. (2023). Endocrine Modulating Chemicals in Food Packaging: A Review of Phthalates and Bisphenols. Compr. Rev. Food Sci. Food Saf..

[15] Luttrell W.E., Baird B.A. (2014). Bisphenol, A. J. Chem. Health Saf..

[16] Manzoor M.F., Tariq T., Fatima B., Sahar A., Tariq F., Munir S., Khan S., Nawaz Ranjha M.M.A., Sameen A., Zeng X.A. (2022). An Insight into Bisphenol A, Food Exposure and Its Adverse Effects on Health: A Review. Front. Nutr..

[17] Ma Y., Liu H., Wu J., Yuan L., Wang Y., Du X., Wang R., Marwa P.W., Petlulu P., Chen X. (2019). The Adverse Health Effects of Bisphenol A and Related Toxicity Mechanisms. Environ. Res..

[18] Liu J., Zhang L., Lu G., Jiang R., Yan Z., Li Y. (2021). Occurrence, Toxicity and Ecological Risk of Bisphenol A Analogues in Aquatic Environment—A Review. Ecotoxicol. Environ. Saf..

[19] Fathy Abdel-satar M., Elgazzar A., Alalfy M., Abdalrashed M., Abdalmageed A., Greash M., Hamed S., Ragab W.S., Moustafa Magdi M. (2023). Correlation Study of Urinary BPA in Women with PCOS. Egypt. J. Forensic Sci. Appl. Toxicol..

[20] Rochester J.R. (2013). Bisphenol A and Human Health: A Review of the Literature. Reprod. Toxicol..

[21] Urbanetz L.A.M.L., Junior J.M.S., Maciel G.A.R., Simões R.d.S., Baracat M.C.P., Baracat E.C. (2023). Does Bisphenol A (BPA) Participates in the Pathogenesis of Polycystic Ovary Syndrome (PCOS)?. Clinics.

[22] Simonelli A., Guadagni R., De Franciscis P., Colacurci N., Pieri M., Basilicata P., Pedata P., Lamberti M., Sannolo N., Miraglia N. (2017). Environmental and Occupational Exposure to Bisphenol A and Endometriosis: Urinary and Peritoneal Fluid Concentration Levels. Int. Arch. Occup. Environ. Health.

[23] Vitku J., Sosvorova L., Chlupacova T., Hampl R., Hill M., Sobotka V., Heracek J., Bicikova M., Starka L. (2015). Differences in Bisphenol A and Estrogen Levels in the Plasma and Seminal Plasma of Men with Different Degrees of Infertility. Physiol. Res..

[24] Presunto M., Mariana M., Lorigo M., Cairrao E. (2023). The Effects of Bisphenol A on Human Male Infertility: A Review of Current Epidemiological Studies. Int. J. Mol. Sci..

[25] Braun J.M., Muckle G., Arbuckle T., Bouchard M.F., Fraser W.D., Ouellet E., Séguin J.R., Oulhote Y., Webster G.M., Lanphear B.P. (2017). Associations of Prenatal Urinary Bisphenol A Concentrations with Child Behaviors and Cognitive Abilities. Environ. Health Perspect..

[26] Welch C., Mulligan K. (2022). Does Bisphenol A Confer Risk of Neurodevelopmental Disorders? What We Have Learned from Developmental Neurotoxicity Studies in Animal Models. Int. J. Mol. Sci..

[27] Kim J.I., Lee Y.A., Shin C.H., Hong Y.C., Kim B.N., Lim Y.H. (2022). Association of Bisphenol A, Bisphenol F, and Bisphenol S with ADHD Symptoms in Children. Environ. Int..

[28] Li C., Sang C., Zhang S., Zhang S., Gao H. (2023). Effects of Bisphenol A and Bisphenol Analogs on the Nervous System. Chin. Med. J..

[29] Sonavane M., Gassman N.R. (2019). Bisphenol A Co-Exposure Effects: A Key Factor in Understanding BPA’s Complex Mechanism and Health Outcomes. Crit. Rev. Toxicol..

[30] Mustieles V., Zhang Y., Yland J., Braun J.M., Williams P.L., Wylie B.J., Attaman J.A., Ford J.B., Azevedo A., Calafat A.M. (2020). Maternal and Paternal Preconception Exposure to Phenols and Preterm Birth. Environ. Int..

[31] Vidal M.S., Menon R., Yu G.F.B., Amosco M.D. (2022). Actions of Bisphenol A on Different Feto-Maternal Compartments Contributing to Preterm Birth. Int. J. Mol. Sci..

[32] Bi J., Wang F., Wei Y., Zhang Y., Jia C., He J., Yao J., Zhang Z., Li Z., Li P. (2022). Association of Serum Bisphenol A Levels with Incident Overweight and Obesity Risk and the Mediating Effect of Adiponectin. Chemosphere.

[33] Deodati A., Bottaro G., Germani D., Carli F., Tait S., Busani L., Della Latta V., Pala A.P., Maranghi F., Tassinari R. (2024). Urinary Bisphenol A and Bis(2-Ethylhexyl) Phthalate Metabolite Concentrations in Children with Obesity: A Case-Control Study. Horm. Res. Paediatr..

[34] Malaisé Y., Lencina C., Cartier C., Olier M., Ménard S., Guzylack-Piriou L. (2020). Perinatal Oral Exposure to Low Doses of Bisphenol A, S or F Impairs Immune Functions at Intestinal and Systemic Levels in Female Offspring Mice. Environ. Health.

[35] Hale A., Moldovan G.L. (2024). Novel Insights into the Role of Bisphenol A (BPA) in Genomic Instability. NAR Cancer.

[36] Buoso E., Masi M., Limosani R.V., Oliviero C., Saeed S., Iulini M., Passoni F.C., Racchi M., Corsini E. (2025). Endocrine Disrupting Toxicity of Bisphenol A and Its Analogs: Implications in the Neuro-Immune Milieu. J. Xenobiotics.

[37] Chen D., Kannan K., Tan H., Zheng Z., Feng Y.L., Wu Y., Widelka M. (2016). Bisphenol Analogues Other Than BPA: Environmental Occurrence, Human Exposure, and Toxicity—A Review. Environ. Sci. Technol..

[38] Food E., Authority S., Fitzgerald R., Loveren V., Civitella C., Castoldi A.F., Bernasconi G. (2020). Assessment of New Information on Bisphenol S (BPS) Submitted in Response to the Decision 1 under REACH Regulation (EC) No 1907/2006. EFSA Support. Publ..

[39] Maćczak A., Cyrkler M., Bukowska B., Michałowicz J. (2017). Bisphenol A, Bisphenol S, Bisphenol F and Bisphenol AF Induce Different Oxidative Stress and Damage in Human Red Blood Cells (in Vitro Study). Toxicol. Vitr..

[40] Ijaz S., Ullah A., Shaheen G., Jahan S. (2020). Exposure of BPA and Its Alternatives like BPB, BPF, and BPS Impair Subsequent Reproductive Potentials in Adult Female Sprague Dawley Rats. Toxicol. Mech. Methods.

[41] Eladak S., Grisin T., Moison D., Guerquin M.J., N’Tumba-Byn T., Pozzi-Gaudin S., Benachi A., Livera G., Rouiller-Fabre V., Habert R. (2015). A New Chapter in the Bisphenol A Story: Bisphenol S and Bisphenol F Are Not Safe Alternatives to This Compound. Fertil. Steril..

[42] Xu J., Huang G., Guo T.L. (2019). Bisphenol S Modulates Type 1 Diabetes Development in Non-Obese Diabetic (NOD) Mice with Diet- and Sex-Related Effects. Toxics.

[43] Hu J., Zhao H., Braun J.M., Zheng T., Zhang B., Xia W., Zhang W., Li J., Zhou Y., Li H. (2019). Associations of Trimester-Specific Exposure to Bisphenols with Size at Birth: A Chinese Prenatal Cohort Study. Environ. Health Perspect..

[44] Kim S., Park E., Park E.K., Lee S., Kwon J.A., Shin B.H., Kang S., Park E.Y., Kim B. (2021). Urinary Concentrations of Bisphenol Mixtures during Pregnancy and Birth Outcomes: The MAKE Study. Int. J. Environ. Res. Public. Health.

[45] Liang F., Huo X., Wang W., Li Y., Zhang J., Feng Y., Wang Y. (2020). Association of Bisphenol A or Bisphenol S Exposure with Oxidative Stress and Immune Disturbance among Unexplained Recurrent Spontaneous Abortion Women. Chemosphere.

[46] Jiang Y., Li J., Xu S., Zhou Y., Zhao H., Li Y., Xiong C., Sun X., Liu H., Liu W. (2020). Prenatal Exposure to Bisphenol A and Its Alternatives and Child Neurodevelopment at 2 Years. J. Hazard. Mater..

[47] George V.C., Rupasinghe H.P.V. (2018). DNA Damaging and Apoptotic Potentials of Bisphenol A and Bisphenol S in Human Bronchial Epithelial Cells. Environ. Toxicol. Pharmacol..

[48] Chen H., Li J., Zhang Y., Zhang W., Li X., Tang H., Liu Y., Li T., He H., Du B. (2022). Bisphenol F Suppresses Insulin-Stimulated Glucose Metabolism in Adipocytes by Inhibiting IRS-1/PI3K/AKT Pathway. Ecotoxicol. Environ. Saf..

[49] Wang Y.X., Liu C., Shen Y., Wang Q., Pan A., Yang P., Chen Y.J., Deng Y.L., Lu Q., Cheng L.M. (2019). Urinary Levels of Bisphenol A, F and S and Markers of Oxidative Stress among Healthy Adult Men: Variability and Association Analysis. Environ. Int..

[50] Usman A., Ikhlas S., Ahmad M. (2019). Occurrence, Toxicity and Endocrine Disrupting Potential of Bisphenol-B and Bisphenol-F: A Mini-Review. Toxicol. Lett..

[51] Jin H., Xie J., Mao L., Zhao M., Bai X., Wen J., Shen T., Wu P. (2020). Bisphenol Analogue Concentrations in Human Breast Milk and Their Associations with Postnatal Infant Growth. Environ. Pollut..

[52] Lee S., An K.S., Kim H.J., Noh H.J., Lee J.W., Lee J., Song K.S., Chae C., Ryu H.Y. (2022). Pharmacokinetics and Toxicity Evaluation Following Oral Exposure to Bisphenol F. Arch. Toxicol..

[53] Odetayo A.F., Adeyemi W.J., Olayaki L.A. (2023). Omega-3 Fatty Acid Ameliorates Bisphenol F-Induced Testicular Toxicity by Modulating Nrf2/NFkB Pathway and Apoptotic Signaling. Front. Endocrinol..

[54] Odetayo A.F., Adeyemi W.J., Olayaki L.A. (2023). In Vivo Exposure to Bisphenol F Induces Oxidative Testicular Toxicity: Role of Erβ and P53/Bcl-2 Signaling Pathway. Front. Reprod. Health.

[55] Ding Z.M., Chen Y.W., Ahmad M.J., Wang Y.S., Yang S.J., Duan Z.Q., Liu M., Yang C.X., Liang A.X., Hua G.H. (2022). Bisphenol F Exposure Affects Mouse Oocyte In Vitro Maturation through Inducing Oxidative Stress and DNA Damage. Environ. Toxicol..

[56] Algonaiman R., Almutairi A.S., Al Zhrani M.M., Barakat H., Algonaiman R., Almutairi A.S., Al Zhrani M.M., Barakat H. (2023). Effects of Prenatal Exposure to Bisphenol A Substitutes, Bisphenol S and Bisphenol F, on Offspring’s Health: Evidence from Epidemiological and Experimental Studies. Biomolecules.

[57] Mu X., Liu J., Wang H., Yuan L., Wang C., Li Y., Qiu J. (2022). Bisphenol F Impaired Zebrafish Cognitive Ability through Inducing Neural Cell Heterogeneous Responses. Environ. Sci. Technol..

[58] Dou L., Sun S., Chen L., Lv L., Chen C., Huang Z., Zhang A., He H., Tao H., Yu M. (2024). The Association between Prenatal Bisphenol F Exposure and Infant Neurodevelopment: The Mediating Role of Placental Estradiol. Ecotoxicol. Environ. Saf..

[59] Gu J., Wu J., Xu S., Zhang L., Fan D., Shi L., Wang J., Ji G. (2020). Bisphenol F Exposure Impairs Neurodevelopment in Zebrafish Larvae (Danio Rerio). Ecotoxicol. Environ. Saf..

[60] Reina-Pérez I., Olivas-Martínez A., Mustieles V., Ruiz-Ojeda F.J., Molina-Molina J.M., Olea N., Fernández M.F. (2021). Bisphenol F and Bisphenol S Promote Lipid Accumulation and Adipogenesis in Human Adipose-Derived Stem Cells. Food Chem. Toxicol..

[61] Wagner V.A., Clark K.C., Carrillo-Sáenz L., Holl K.A., Velez-Bermudez M., Simonsen D., Grobe J.L., Wang K., Thurman A., Solberg Woods L.C. (2021). Bisphenol F Exposure in Adolescent Heterogeneous Stock Rats Affects Growth and Adiposity. Toxicol. Sci..

[62] Jacobson M.H., Woodward M., Bao W., Liu B., Trasande L. (2019). Urinary Bisphenols and Obesity Prevalence Among U.S. Children and Adolescents. J. Endocr. Soc..

[63] Rancière F., Botton J., Slama R., Lacroix M.Z., Debrauwer L., Charles M.A., Roussel R., Balkau B., Magliano D.J., Balkau B. (2019). Exposure to Bisphenol A and Bisphenol s and Incident Type 2 Diabetes: A Case-Cohort Study in the French Cohort D.E.S.I.R. Environ. Health Perspect..

[64] Yuan L., Qian L., Qian Y., Liu J., Yang K., Huang Y., Wang C., Li Y., Mu X. (2019). Bisphenol F-Induced Neurotoxicity toward Zebrafish Embryos. Environ. Sci. Technol..

[65] Wang H., Qi S., Mu X., Yuan L., Li Y., Qiu J. (2022). Bisphenol F Induces Liver-Gut Alteration in Zebrafish. Sci. Total Environ..

[66] Lu Y., Chen S., Jin H., Tang L., Xia M. (2023). Associations of Bisphenol F and S, as Substitutes for Bisphenol A, with Cardiovascular Disease in American Adults. J. Appl. Toxicol..

[67] García-Recio E., Costela-Ruiz V.J., Illescas-Montes R., Melguizo-Rodríguez L., García-Martínez O., Ruiz C., De Luna-Bertos E. (2023). Modulation of Osteogenic Gene Expression by Human Osteoblasts Cultured in the Presence of Bisphenols BPF, BPS, or BPAF. Int. J. Mol. Sci..

[68] Catenza C.J., Farooq A., Shubear N.S., Donkor K.K. (2021). A Targeted Review on Fate, Occurrence, Risk and Health Implications of Bisphenol Analogues. Chemosphere.

[69] Escrivá L., Zilliacus J., Hessel E., Beronius A. (2021). Assessment of the Endocrine Disrupting Properties of Bisphenol AF: A Case Study Applying the European Regulatory Criteria and Guidance. Environ. Health.

[70] Lu S., Liu M., Liu H., Yang C., Zhu J., Ling Y., Kuang H. (2024). Gestational Exposure to Bisphenol AF Causes Endocrine Disorder of Corpus Luteum by Altering Ovarian SIRT-1/Nrf2/NF-KB Expressions and Macrophage Proangiogenic Function in Mice. Biochem. Pharmacol..

[71] Zhan W., Tang W., Shen X., Xu H., Zhang J. (2023). Exposure to Bisphenol A and Its Analogs and Polycystic Ovarian Syndrome in Women of Childbearing Age: A Multicenter Case-Control Study. Chemosphere.

[72] Yue H., Yang X., Wu X., Tian Y., Xu P., Sang N. (2023). Identification of Risk for Ovarian Disease Enhanced by BPB or BPAF Exposure. Environ. Pollut..

[73] Wu X., Yang X., Tian Y., Xu P., Yue H., Sang N. (2023). Bisphenol B and Bisphenol AF Exposure Enhances Uterine Diseases Risks in Mouse. Environ. Int..

[74] Meng X., Su S., Wei X., Wang S., Guo T., Li J., Song H., Wang M., Wang Z. (2023). Exposure to Bisphenol A Alternatives Bisphenol AF and Fluorene-9-Bisphenol Induces Gonadal Injuries in Male Zebrafish. Ecotoxicol. Environ. Saf..

[75] Xue S., Liu L., Dong M., Xue W., Zhou S., Li X., Guo S., Yan W. (2023). Prenatal Exposure to Bisphenol AF Induced Male Offspring Reproductive Dysfunction by Triggering Testicular Innate and Adaptive Immune Responses. Ecotoxicol. Environ. Saf..

[76] Wang L., Zhu Y., Gu J., Yin X., Guo L., Qian L., Shi L., Guo M., Ji G. (2023). The Toxic Effect of Bisphenol AF and Nanoplastic Coexposure in Parental and Offspring Generation Zebrafish. Ecotoxicol. Environ. Saf..

[77] Wu X., Li S., Ni Y., Qi C., Bai S., Xu Q., Fan Y., Ma X., Lu C., Du G. (2023). Maternal BPAF Exposure Impaired Synaptic Development and Caused Behavior Abnormality in Offspring. Ecotoxicol. Environ. Saf..

[78] Yang Q., Liu J., Ding J., Liu J. (2023). Neurodevelopmental Toxicity of Bisphenol AF in Zebrafish Larvae and the Protective Effects of Curcumin. J. Appl. Toxicol..

[79] Chernis N., Masschelin P., Cox A.R., Hartig S.M. (2020). Bisphenol AF Promotes Inflammation in Human White Adipocytes. Am. J. Physiol. Cell Physiol..

[80] Chen M., Lv C., Zhang S., Tse L.A., Hong X., Liu X., Ding Y., Xiao P., Tian Y., Gao Y. (2023). Bisphenol A Substitutes and Childhood Obesity at 7 Years: A Cross-Sectional Study in Shandong, China. Environ. Sci. Pollut. Res..

[81] Cui M., Tzioufa F., Bruton J., Westerblad H., Munic Kos V. (2025). The Impact of Bisphenol AF on Skeletal Muscle Function and Differentiation in Vitro. Toxicology.

[82] Gyimah E., Zhu X., Zhang Z., Guo M., Xu H., Mensah J.K., Dong X., Zhang Z., Gyimah G.N.W. (2022). Oxidative Stress and Apoptosis in Bisphenol AF–Induced Neurotoxicity in Zebrafish Embryos. Environ. Toxicol. Chem..

[83] Hunt P.R. (2017). The *C. elegans* Model in Toxicity Testing. J. Appl. Toxicol..

[84] Hartung T. (2009). Toxicology for the Twenty-First Century. Nature.

[85] Hunt P.R., Camacho J.A., Sprando R.L. (2020). *Caenorhabditis elegans* for Predictive Toxicology. Curr. Opin. Toxicol..

[86] Poh W.T., Stanslas J. (2024). The New Paradigm in Animal Testing—“3Rs Alternatives”. Regul. Toxicol. Pharmacol..

[87] Ha N.M., Tran S.H., Shim Y.H., Kang K. (2022). *Caenorhabditis elegans* as a Powerful Tool in Natural Product Bioactivity Research. Appl. Biol. Chem..

[88] Wang Y., Ezemaduka A.N., Tang Y., Chang Z. (2009). Understanding the Mechanism of the Dormant Dauer Formation of *C. elegans*: From Genetics to Biochemistry. IUBMB Life.

[89] Mata-Cabana A., Romero-Expósito F.J., Geibel M., Piubeli F.A., Merrow M., Olmedo M. (2022). Deviations from Temporal Scaling Support a Stage-Specific Regulation for *C. elegans* Postembryonic Development. BMC Biol..

[90] Muschiol D., Schroeder F., Traunspurger W. (2009). Life Cycle and Population Growth Rate of *Caenorhabditis elegans* Studied by a New Method. BMC Ecol..

[91] Indong R.A., Park J.M., Hong J.K., Lyou E.S., Han T., Hong J.K., Lee T.K., Lee J.I. (2024). A Simple Protocol for Cultivating the Bacterivorous Soil Nematode *Caenorhabditis elegans* in Its Natural Ecology in the Laboratory. Front. Microbiol..

[92] *C. elegans* Sequencing Consortium (1998). Genome Sequence of the Nematode *C. elegans*: A Platform for Investigating Biology. Science.

[93] Leung M.C.K., Williams P.L., Benedetto A., Au C., Helmcke K.J., Aschner M., Meyer J.N. (2008). *Caenorhabditis elegans*: An Emerging Model in Biomedical and Environmental Toxicology. Toxicol. Sci..

[94] Baumeister R., Ge L. (2002). The Worm in Us—*Caenorhabditis elegans* as a Model of Human Disease. Trends Biotechnol..

[95] Williams D.C., Bailey D.C., Fitsanakis V.A. (2017). *Caenorhabditis elegans* as a Model to Assess Reproductive and Developmental Toxicity. Reproductive and Developmental Toxicology.

[96] Li Y., Zhong L., Zhang L., Shen X., Kong L., Wu T. (2021). Research Advances on the Adverse Effects of Nanomaterials in a Model Organism, *Caenorhabditis elegans*. Environ. Toxicol. Chem..

[97] Melnikov K., Kucharíková S., Bárdyová Z., Botek N., Kaiglová A. (2023). Applications of a Powerful Model Organism *Caenorhabditis elegans* to Study the Neurotoxicity Induced by Heavy Metals and Pesticides. Physiol. Res..

[98] Istiban M.N., De Fruyt N., Kenis S., Beets I. (2024). Evolutionary Conserved Peptide and Glycoprotein Hormone-like Neuroendocrine Systems in *C. elegans*. Mol. Cell Endocrinol..

[99] Fielenbach N., Antebi A. (2008). *C. elegans* Dauer Formation and the Molecular Basis of Plasticity. Genes. Dev..

[100] Mishra S., Dabaja M., Akhlaq A., Pereira B., Marbach K., Rovcanin M., Chandra R., Caballero A., Fernandes de Abreu D., Ch’ng Q.L. (2023). Specific Sensory Neurons and Insulin-like Peptides Modulate Food Type-Dependent Oogenesis and Fertilization in *Caenorhabditis elegans*. Elife.

[101] Zečić A., Braeckman B.P. (2020). DAF-16/FoxO in *Caenorhabditis elegans* and Its Role in Metabolic Remodeling. Cells.

[102] Gerisch B., Rottiers V., Li D., Motola D.L., Cummins C.L., Lehrach H., Mangelsdorf D.J., Antebi A. (2007). A Bile Acid-like Steroid Modulates *Caenorhabditis elegans* Lifespan through Nuclear Receptor Signaling. Proc. Natl. Acad. Sci. USA.

[103] Queirós L., Pereira J.L., Gonçalves F.J.M., Pacheco M., Aschner M., Pereira P. (2019). *Caenorhabditis elegans* as a Tool for Environmental Risk Assessment: Emerging and Promising Applications for a “Nobelized Worm”. Crit. Rev. Toxicol..

[104] Long N.P., Kang J.S., Kim H.M. (2023). *Caenorhabditis elegans*: A Model Organism in the Toxicity Assessment of Environmental Pollutants. Environ. Sci. Pollut. Res..

[105] Yu Y., Chen H., Hua X., Wang C., Dong C., Xie D., Tan S., Xiang M., Li H. (2022). A Review of the Reproductive Toxicity of Environmental Contaminants in *Caenorhabditis elegans*. Hyg. Environ. Health Adv..

[106] Li Y., Gao S., Jing H., Qi L., Ning J., Tan Z., Yang K., Zhao C., Ma L., Li G. (2013). Correlation of Chemical Acute Toxicity between the Nematode and the Rodent. Toxicol. Res..

[107] Zhuang Z., Zhao Y., Wu Q., Li M., Liu H., Sun L., Gao W., Wang D. (2014). Adverse Effects from Clenbuterol and Ractopamine on Nematode *Caenorhabditis elegans* and the Underlying Mechanism. PLoS ONE.

[108] Amrit F.R.G., Ratnappan R., Keith S.A., Ghazi A. (2014). The *C. elegans* Lifespan Assay Toolkit. Methods.

[109] Ficociello G., Gerardi V., Uccelletti D., Setini A. (2021). Molecular and Cellular Responses to Short Exposure to Bisphenols A, F, and S and Eluates of Microplastics in *C. elegans*. Environ. Sci. Pollut. Res..

[110] García-Espiñeira M.C., Tejeda-Benítez L.P., Olivero-Verbel J. (2018). Toxic Effects of Bisphenol A, Propyl Paraben, and Triclosan on *Caenorhabditis elegans*. Int. J. Environ. Res. Public Health.

[111] Hornos Carneiro M.F., Shin N., Karthikraj R., Barbosa F., Kannan K., Colaiácovo M.P. (2020). Antioxidant CoQ10 Restores Fertility by Rescuing Bisphenol A-Induced Oxidative DNA Damage in the *Caenorhabditis elegans* Germline. Genetics.

[112] Zhou D., Yang J., Li H., Cui C., Yu Y., Liu Y., Lin K. (2016). The Chronic Toxicity of Bisphenol A to *Caenorhabditis elegans* after Long-Term Exposure at Environmentally Relevant Concentrations. Chemosphere.

[113] Hamamba N., Jie L., Wei Z.X., Qin Z.C., SARPONG F., Hua Z.X., HAMAMBA N., Jie L., Wei Z.X., Qin Z.C. (2020). Effects of Treatment with Different Combinations of Bisphenol Compounds on the Mortality of *Caenorhabditis elegans*. Biomed. Environ. Sci..

[114] Wang D. (2019). Avoidance Behavior of Nematodes to Environmental Toxicants or Stresses. Target Organ Toxicology in Caenorhabditis elegans.

[115] Wang M., Nie Y., Liu Y., Dai H., Wang J., Si B., Yang Z., Cheng L., Liu Y., Chen S. (2019). Transgenerational Effects of Diesel Particulate Matter on *Caenorhabditis elegans* through Maternal and Multigenerational Exposure. Ecotoxicol. Environ. Saf..

[116] De la Parra-Guerra A., Olivero-Verbel J. (2020). Toxicity of Nonylphenol and Nonylphenol Ethoxylate on *Caenorhabditis elegans*. Ecotoxicol. Environ. Saf..

[117] Xiao X., Zhang X., Zhang C., Li J., Zhao Y., Zhu Y., Zhang J., Zhou X. (2019). Toxicity and Multigenerational Effects of Bisphenol S Exposure to *Caenorhabditis elegans* on Developmental, Biochemical, Reproductive and Oxidative Stress. Toxicol. Res..

[118] Hyun M., Rathor L., Kim H.J., McElroy T., Hwang K.H., Wohlgemuth S., Curry S., Xiao R., Leeuwenburgh C., Heo J.D. (2021). Comparative Toxicities of BPA, BPS, BPF, and TMBPF in the Nematode *Caenorhabditis elegans* and Mammalian Fibroblast Cells. Toxicology.

[119] Kucharíková S., Hockicková P., Melnikov K., Bárdyová Z., Kaiglová A. (2023). The *Caenorhabditis elegans* Cuticle Plays an Important Role against Toxicity to Bisphenol A and Bisphenol S. Toxicol. Rep..

[120] Kaiglová A., Bárdyová Z., Hockicková P., Zvolenská A., Melnikov K., Kucharíková S. (2025). *Caenorhabditis elegans* as a Model to Assess the Potential Risk to Human Health Associated with the Use of Bisphenol A and Its Substitutes. Int. J. Mol. Sci..

[121] Liu F., Zhang Y., Zhang M., Luo Q., Cao X., Cui C., Lin K., Huang K. (2020). Toxicological Assessment and Underlying Mechanisms of Tetrabromobisphenol A Exposure on the Soil Nematode *Caenorhabditis elegans*. Chemosphere.

[122] Wang D., Wang Y., Shen L. (2010). Confirmation of Combinational Effects of Calcium with Other Metals in a Paper Recycling Mill Effluent on Nematode Lifespan with Toxicity Identification Evaluation Method. J. Environ. Sci..

[123] Scharf A., Pohl F., Egan B.M., Kocsisova Z., Kornfeld K. (2021). Reproductive Aging in *Caenorhabditis elegans*: From Molecules to Ecology. Front. Cell Dev. Biol..

[124] Corsi A.K., Golden A., Conway S.J., Camacho J.A., Welch B., Sprando R.L., Hunt P.R. (2023). Reproductive-Toxicity-Related Endpoints in *C. elegans* Are Consistent with Reduced Concern for Dimethylarsinic Acid Exposure Relative to Inorganic Arsenic. J. Dev. Biol..

[125] Wang M., Chen Y., Kickhoefer V.A., Rome L.H., Allard P., Mahendra S. (2019). A Vault-Encapsulated Enzyme Approach for Efficient Degradation and Detoxification of Bisphenol A and Its Analogues. ACS Sustain. Chem. Eng..

[126] Allard P., Colaiácovo M.P. (2010). Bisphenol A Impairs the Double-Strand Break Repair Machinery in the Germline and Causes Chromosome Abnormalities. Proc. Natl. Acad. Sci. USA.

[127] Chen Y., Shu L., Qiu Z., Lee D.Y., Settle S.J., Que Hee S., Telesca D., Yang X., Allard P. (2016). Exposure to the BPA-Substitute Bisphenol S Causes Unique Alterations of Germline Function. PLoS Genet..

[128] Yu Y., Hua X., Chen H., Yang Y., Dang Y., Xiang M. (2022). Tetrachlorobisphenol A Mediates Reproductive Toxicity in *Caenorhabditis elegans* via DNA Damage-Induced Apoptosis. Chemosphere.

[129] Roberts A.H., Bowen J.E., Zhou X., Burke I., Wenaas M.H., Blake T.A., Timmons S.C., Kuzmanov A. (2022). Synthesis and Reproductive Toxicity of Bisphenol A Analogs with Cyclic Side Chains in *Caenorhabditis elegans*. Toxicol. Ind. Health.

[130] Rathor L., Lee H.J., McElroy T., Beck S., Bailey J., Wohlgemuth S., Kim S.-H., Heo J., Xiao R., Han S.M. (2024). Bisphenol TMC Disturbs Mitochondrial Activity and Biogenesis, Reducing Lifespan and Healthspan in the Nematode *Caenorhabditis elegans*. bioRxiv.

[131] Park H.E.H., Jung Y., Lee S.J.V. (2017). Survival Assays Using *Caenorhabditis elegans*. Mol. Cells.

[132] Jin L., Dou T.T., Chen J.Y., Duan M.X., Zhen Q., Wu H.Z., Zhao Y.L. (2022). Sublethal Toxicity of Graphene Oxide in *Caenorhabditis elegans* under Multi-Generational Exposure. Ecotoxicol. Environ. Saf..

[133] Ayuda-Durán B., Garzón-García L., González-Manzano S., Santos-Buelga C., González-Paramás A.M. (2024). Insights into the Neuroprotective Potential of Epicatechin: Effects against Aβ-Induced Toxicity in *Caenorhabditis elegans*. Antioxidants.

[134] Kim S.W., Moon J., An Y.J. (2018). Matricidal Hatching Can Induce Multi-Generational Effects in Nematode *Caenorhabditis elegans* after Dietary Exposure to Nanoparticles. Environ. Sci. Pollut. Res..

[135] Zhao Y., Chen J., Wang R., Pu X., Wang D. (2023). A Review of Transgenerational and Multigenerational Toxicology in the in Vivo Model Animal *Caenorhabditis elegans*. J. Appl. Toxicol..

[136] Camacho J., Truong L., Kurt Z., Chen Y.W., Morselli M., Gutierrez G., Pellegrini M., Yang X., Allard P. (2018). The Memory of Environmental Chemical Exposure in *C. elegans* Is Dependent on the Jumonji Demethylases Jmjd-2 and Jmjd-3/Utx-1. Cell Rep..

[137] McDonough C.M., Guo D.J., Guo T.L. (2021). Developmental Toxicity of Bisphenol S in *Caenorhabditis elegans* and NODEF Mice. Neurotoxicology.

[138] Liu F., Luo Q., Zhang Y., Huang K., Cao X., Cui C., Lin K., Zhang M. (2020). Trans-Generational Effect of Neurotoxicity and Related Stress Response in *Caenorhabditis elegans* Exposed to Tetrabromobisphenol A. Sci. Total Environ..

[139] Liu F., Cao X., Tian F., Jiang J., Lin K., Cheng J., Hu X. (2023). Continuous and Discontinuous Multi-Generational Disturbances of Tetrabromobisphenol A on Longevity in *Caenorhabditis elegans*. Ecotoxicol. Environ. Saf..

[140] Wang D., Xing X. (2008). Assessment of Locomotion Behavioral Defects Induced by Acute Toxicity from Heavy Metal Exposure in Nematode *Caenorhabditis elegans*. J. Environ. Sci..

[141] Zhang S., Li F., Zhou T., Wang G., Li Z. (2020). *Caenorhabditis elegans* as a Useful Model for Studying Aging Mutations. Front. Endocrinol..

[142] Zheng F., Chen C., Aschner M. (2022). Neurotoxicity Evaluation of Nanomaterials Using *C. elegans*: Survival, Locomotion Behaviors, and Oxidative Stress. Curr. Protoc..

[143] Wang Y., Gai T., Zhang L., Chen L., Wang S., Ye T., Zhang W. (2023). Neurotoxicity of Bisphenol A Exposure on *Caenorhabditis elegans* Induced by Disturbance of Neurotransmitter and Oxidative Damage. Ecotoxicol. Environ. Saf..

[144] Buckingham S.D., Sattelle D.B. (2009). Fast, Automated Measurement of Nematode Swimming (Thrashing) without Morphometry. BMC Neurosci..

[145] Petratou D., Fragkiadaki P., Lionaki E., Tavernarakis N. (2023). Assessing Locomotory Rate in Response to Food for the Identification of Neuronal and Muscular Defects in *C. elegans*. STAR Protoc..

[146] Hering I., Le D.T., von Mikecz A. (2022). How to Keep up with the Analysis of Classic and Emerging Neurotoxins: Age-Resolved Fitness Tests in the Animal Model *Caenorhabditis elegans*—A Step-by-Step Protocol. EXCLI J..

[147] Liu Y., Zhang W., Wang Y., Liu H., Zhang S., Ji X., Qiao K. (2022). Oxidative Stress, Intestinal Damage, and Cell Apoptosis: Toxicity Induced by Fluopyram in *Caenorhabditis elegans*. Chemosphere.

[148] Zhang H., Gao S., Chen W. (2022). Automated Recognition and Analysis of Head Thrashes Behavior in *C. elegans*. BMC Bioinform..

[149] Tan L., Wang S., Wang Y., He M., Liu D. (2015). Bisphenol A Exposure Accelerated the Aging Process in the Nematode *Caenorhabditis elegans*. Toxicol. Lett..

[150] Zhao K., Zhang Y., Liu M., Huang Y., Wang S., An J., Wang Y., Shang Y. (2023). The Joint Effects of Nanoplastics and TBBPA on Neurodevelopmental Toxicity in *Caenorhabditis elegans*. Toxicol. Res..

[151] Yu Y., Tan S., Guo H., Hua X., Chen H., Yang Y., Xie D., Yi C., Ling H., Xiang M. (2024). Chronic Neurotoxicity of Tetrabromobisphenol A: Induction of Oxidative Stress and Damage to Neurons in *Caenorhabditis elegans*. Chemosphere.

[152] Margie O., Palmer C., Chin-Sang I. (2013). *C. elegans* Chemotaxis Assay. J. Vis. Exp..

[153] Hopewell H., Floyd K.G., Burnell D., Hancock J.T., Allainguillaume J., Ladomery M.R., Wilson I.D. (2017). Residual Ground-Water Levels of the Neonicotinoid Thiacloprid Perturb Chemosensing of *Caenorhabditis elegans*. Ecotoxicology.

[154] Crombie T.A., Chikuturudzi C., Cook D.E., Andersen E.C. (2022). An Automated Approach to Quantify Chemotaxis Index in *C. elegans*. Micro. Publ. Biol.

[155] Gao X., Yu J., Zhang L., Shi H., Yan Y., Han Y., Fang M., Liu Y., Wu C., Fan S. (2024). Mulberrin Extends Lifespan in *Caenorhabditis elegans* through Detoxification Function. J. Appl. Toxicol..

[156] Ijomone O.M., Gubert P., Okoh C.O.A., Varão A.M., Amaral L.d.O., Aluko O.M., Aschner M. (2021). Application of Fluorescence Microscopy and Behavioral Assays to Demonstrating Neuronal Connectomes and Neurotransmitter Systems in *C. elegans*. Neuromethods.

[157] Gaur A.V., Agarwal R. (2021). Risperidone Induced Alterations in Feeding and Locomotion Behavior of *Caenorhabditis elegans*. Curr. Res. Toxicol..

[158] Boyd W.A., McBride S.J., Freedman J.H. (2007). Effects of Genetic Mutations and Chemical Exposures on *Caenorhabditis elegans* Feeding: Evaluation of a Novel, High-Throughput Screening Assay. PLoS ONE.

[159] Tejeda-Benitez L., Olivero-Verbel J. (2016). *Caenorhabditis elegans*, a Biological Model for Research in Toxicology. Rev. Environ. Contam. Toxicol..

[160] Kohra S., Kuwahara K., Takao Y., Ishibashi Y., Ho C.L., Arizono K., Tominagae N. (2002). Effect of Bisphenol A on the Feeding Behavior of *Caenorhabditis elegans*. J. Health Sci..

[161] Yu Y., Chen H., Hua X., Dang Y., Han Y., Yu Z., Chen X., Ding P., Li H. (2020). Polystyrene Microplastics (PS-MPs) Toxicity Induced Oxidative Stress and Intestinal Injury in Nematode *Caenorhabditis elegans*. Sci. Total Environ..

[162] Wang F., Jin T., Li H., Long H., Liu Y., Jin S., Lu Y., Peng Y., Liu C., Zhao L. (2023). Cannabidivarin Alleviates α-Synuclein Aggregation via DAF-16 in *Caenorhabditis elegans*. FASEB J..

[163] Imanikia S., Galea F., Nagy E., Phillips D.H., Stürzenbaum S.R., Arlt V.M. (2016). The Application of the Comet Assay to Assess the Genotoxicity of Environmental Pollutants in the Nematode *Caenorhabditis elegans*. Environ. Toxicol. Pharmacol..

[164] Bradford B.R., Briand N.E., Fassnacht N., Gervasio E.D., Nowakowski A.M., FitzGibbon T.C., Maurina S., Benjamin A.V., Kelly M.E., Checchi P.M. (2020). Counteracting Environmental Chemicals with Coenzyme Q10: An Educational Primer for Use with “Antioxidant CoQ10 Restores Fertility by Rescuing Bisphenol A-Induced Oxidative DNA Damage in the *Caenorhabditis elegans* Germline”. Genetics.

[165] Zhou D. (2018). Ecotoxicity of Bisphenol S to *Caenorhabditis elegans* by Prolonged Exposure in Comparison with Bisphenol A. Environ. Toxicol. Chem..

[166] Shin N., Cuenca L., Karthikraj R., Kannan K., Colaiácovo M.P. (2019). Assessing Effects of Germline Exposure to Environmental Toxicants by High-Throughput Screening in *C. elegans*. PLoS Genet..

[167] Yang Y., Li M., Zheng J., Zhang D., Ding Y., Yu H.Q. (2024). Environmentally Relevant Exposure to Tetrabromobisphenol a Induces Reproductive Toxicity via Regulating Glucose-6-Phosphate 1-Dehydrogenase and Sperm Activation in *Caenorhabditis elegans*. Sci. Total Environ..

[168] Qu M., Qiu Y., Kong Y., Wang D. (2019). Amino Modification Enhances Reproductive Toxicity of Nanopolystyrene on Gonad Development and Reproductive Capacity in Nematode *Caenorhabditis elegans*. Environ. Pollut..

[169] Germoglio M., Adamo A., Incerti G., Cartenì F., Gigliotti S., Storlazzi A., Mazzoleni S. (2022). Self-DNA Exposure Induces Developmental Defects and Germline DNA Damage Response in *Caenorhabditis elegans*. Biology.

[170] Bradford B.R., Whidden E., Gervasio E.D., Checchi P.M., Raley-Susman K.M. (2020). Neonicotinoid-Containing Insecticide Disruption of Growth, Locomotion, and Fertility in *Caenorhabditis elegans*. PLoS ONE.

[171] Moreman J., Lee O., Trznadel M., David A., Kudoh T., Tyler C.R. (2017). Acute Toxicity, Teratogenic, and Estrogenic Effects of Bisphenol A and Its Alternative Replacements Bisphenol S, Bisphenol F, and Bisphenol AF in Zebrafish Embryo-Larvae. Environ. Sci. Technol..

[172] Weinhouse C., Truong L., Meyer J.N., Allard P. (2018). *Caenorhabditis elegans* as an Emerging Model System in Environmental Epigenetics. Environ. Mol. Mutagen..

[173] Harlow P.H., Perry S.J., Stevens A.J., Flemming A.J. (2018). Comparative Metabolism of Xenobiotic Chemicals by Cytochrome P450s in the Nematode *Caenorhabditis elegans*. Sci. Rep..

[174] Hunt P.R., Olejnik N., Sprando R.L. (2012). Toxicity Ranking of Heavy Metals with Screening Method Using Adult *Caenorhabditis elegans* and Propidium Iodide Replicates Toxicity Ranking in Rat. Food Chem. Toxicol..

[175] Ruszkiewicz J.A., Pinkas A., Miah M.R., Weitz R.L., Lawes M.J.A., Akinyemi A.J., Ijomone O.M., Aschner M. (2018). *C. elegans* as a Model in Developmental Neurotoxicology. Toxicol. Appl. Pharmacol..

[176] Wu T., Xu H., Liang X., Tang M. (2019). *Caenorhabditis elegans* as a Complete Model Organism for Biosafety Assessments of Nanoparticles. Chemosphere.

[177] Mimoto A., Fujii M., Usami M., Shimamura M., Hirabayashi N., Kaneko T., Sasagawa N., Ishiura S. (2007). Identification of an Estrogenic Hormone Receptor in *Caenorhabditis elegans*. Biochem. Biophys. Res. Commun..

[178] Giunti S., Andersen N., Rayes D., De Rosa M.J. (2021). Drug Discovery: Insights from the Invertebrate *Caenorhabditis elegans*. Pharmacol. Res. Perspect..

[179] Buoso E., Kenda M., Masi M., Linciano P., Galbiati V., Racchi M., Dolenc M.S., Corsini E. (2021). Effects of Bisphenols on RACK1 Expression and Their Immunological Implications in THP-1 Cells. Front. Pharmacol..

[180] Pahović P.Š., Iulini M., Maddalon A., Galbiati V., Buoso E., Dolenc M.S., Corsini E. (2023). In Vitro Effects of Bisphenol Analogs on Immune Cells Activation and Th Differentiation. Endocr. Metab. Immune Disord. Drug Targets.

[181] Lambré C., Barat Baviera J.M., Bolognesi C., Chesson A., Cocconcelli P.S., Crebelli R., Gott D.M., Grob K., Lampi E., Mengelers M. (2023). Re-Evaluation of the Risks to Public Health Related to the Presence of Bisphenol A (BPA) in Foodstuffs. EFSA J..

[182] Benian G.M., Epstein H.F. (2011). *Caenorhabditis elegans* Muscle: A Genetic and Molecular Model for Protein Interactions in the Heart. Circ. Res..

[183] McGary K.L., Park T.J., Woods J.O., Cha H.J., Wallingford J.B., Marcotte E.M. (2010). Systematic Discovery of Nonobvious Human Disease Models through Orthologous Phenotypes. Proc. Natl. Acad. Sci. USA.

[184] Xiong H., Pears C., Woollard A. (2017). An Enhanced *C. elegans* Based Platform for Toxicity Assessment. Sci. Rep..

